# The effect of synbiotics supplementation on anthropometric indicators and lipid profiles in women with polycystic ovary syndrome: a randomized controlled trial

**DOI:** 10.1186/s12944-020-01244-4

**Published:** 2020-04-06

**Authors:** Elham Karimi, Javad Heshmati, Nooshin Shirzad, Samira Vesali, Mohammad Javad Hosseinzadeh-Attar, Ashraf Moini, Mahdi Sepidarkish

**Affiliations:** 1 Department of Clinical Nutrition, School of Nutrition and Food Science, Food Security Research Center, Isfahan University of Medical Sciences, Isfahan, Iran; 2 Research Development Center, Arash Women’s Hospital, Tehran University of Medical Sciences, Tehran, Iran; 3 Department of Nutritional Science, School of Nutritional Science and Food Technology, Kermanshah University of Medical Sciences, Kermanshah, Iran; 4 Endocrinology and Metabolism Research Center, Endocrinology and Metabolism Clinical Sciences Institute, Tehran University of Medical Sciences, Tehran, Iran; 5 Reproductive Epidemiology Research Center, Royan Institute for Reproductive Biomedicine, ACECR, Tehran, Iran; 6 Department of Interdisciplinary Research in Diabetes, Obesity and Metabolism, Reproductive Biomedicine Research Center, Royan Institute for Reproductive Biomedicine, ACECR, Tehran, Iran; 7 Department of Clinical Nutrition, School of Nutritional Sciences and Dietetics, International Campus, Tehran University of Medical Sciences (IC-TUMS), Tehran, Iran; 8 Discipline of Medicine, Centre of Research Excellence in Translating Nutritional Science to Good Health, Royal Adelaide Hospital, University of Adelaide, Adelaide, Australia; 9 Department of Gynecology and Obstetrics, Roointan-Arash Maternity Hospital, Tehran University of Medical Sciences, Tehran, Iran; 10 Department of Biostatistics and Epidemiology, School of Public Health, Babol University of Medical Sciences, Babol, Iran

**Keywords:** Polycystic ovary syndrome, Synbiotics, Cholesterol, LDL, Cholesterol, HDL, Triglycerides

## Abstract

**Background:**

Different therapies have been suggested for polycystic ovary syndrome (PCOS), but changes in lifestyle and diet have been considered. Diet and dietary factors can be very effective in modifying the disease. The positive effects of probiotic and synbiotics supplementation on improving lipid profiles and anthropometric indices have been examined in various diseases. This study was conducted to evaluate the effects of synbiotics supplementation on lipid and anthropometric profiles in infertile women with PCOS.

**Methods:**

PCOS patients aged 19–37 years old were randomized to receive either synbiotics supplement (*n* = 50) or placebo (*n* = 49) for 12 weeks.

**Results:**

Consumption of synbiotics compared to the placebo, resulted in a significant decrease in Low-density lipoprotein cholesterol (LDL) value (Change Mean Difference (CMD): 4.66, 95%CI: 0.20, 9.13) and a significant increase in high-density lipoprotein cholesterol (HDL) (CMD: 1.80, 95%CI: 0.34, 3.26). Although we failed to find a significant effect of synbiotics consumption on total cholesterol (TC) and triglyceride (TG) levels. We did not find differences in anthropometric indices between groups.

**Conclusions:**

Overall, 12 weeks of synbiotics supplementation among PCOS women resulted in beneficial effects on LDL and HDL, although it is not yet clear how much our findings are clinically significant and more clinical studies with larger sample sizes are still needed.

**Trial registration:**

Iranian Registry of clinical Trial, IRCT.ir, ID: IRCT2014110515536N2. Registered on 19 December 2015.

**Graphical abstract:**

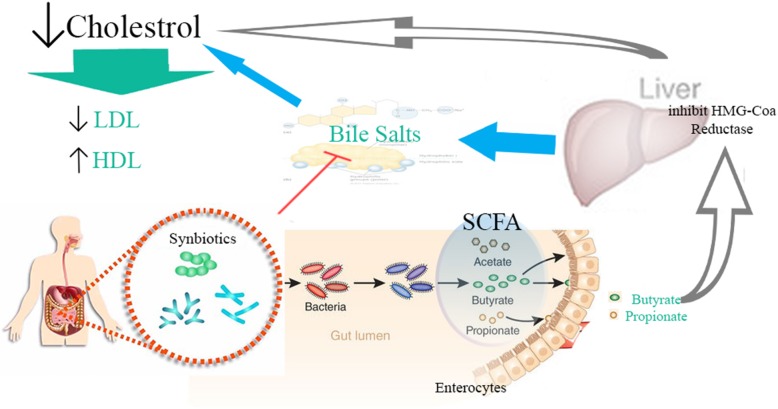

## Background

PCOS is one of the most common polygenic endocrine disorders in women of reproductive age [1, 2]. The prevalence of this syndrome varies in different countries [3]. It may be influenced by race and body composition [4]. Based on the Rotterdam consensus, the prevalence of women with PCOS is more than 15% [5–7]. It is over 30% in women with overweight and obesity [4, 6]. There is a great deal of cost on health systems resulting from complications associated with PCOS [8]. More than half of the patients with PCOS are obese. Obesity increases the risk of diabetes mellitus and cardiovascular diseases [9]. Insulin resistance and increased serum insulin are commonly found in PCOS. One third of women with PCOS have impaired glucose tolerance and 10–70% of them are with type 2 diabetes [10]. One of other complications in PCOS is abnormalities in the metabolism of lipoproteins, which includes increased cholesterol, TG, and LDL and decreased HDL [11]. Various studies have shown that correction of the lipid pattern in patients with PCOS can be very helpful [12]. Different therapies have been suggested for PCOS, but changes in lifestyle and diet have been considered [13]. Diet and dietary factors can be very effective in modifying the disease. Probiotics and prebiotics dietary are considerations [14, 15].

Probiotics are living microorganisms originate from the gastrointestinal tract. They affect the human’s metabolic and inflammatory indices. Prebiotics are indigestible food compounds that selectively stimulate the growth and activity of some bacteria in intestinal flora. Synbiotics is a blend of prebiotic food and probiotic bacteria [16, 17]. Animal studies have shown that intestinal bacterial flora changes in PCOS and goes to pathogenic bacteria [18]. The positive effects of probiotic and synbiotics supplementation on improving lipid profiles and anthropometric indices have been examined in various diseases [19, 20]. This study was conducted to evaluate the effects of synbiotics supplementation on lipid and anthropometric profiles in infertile women with PCOS.

## Material and methods

### Design of the trial

This was a double blind placebo controlled trial conducted at the Arash hospital, Tehran, Iran. Women with PCOS were allocated to receive synbiotics supplement (*n* = 44) or placebo (n = 44) in a 1:1 ratio for a period of 12 weeks. The study protocol was approved by the Tehran University of Medical Sciences Ethics Committee, which granted full ethical approval for the trial in September 2015. This trial was registered at Iranian Registry of clinical Trial (www.IRCT.ir) by the number of IRCT2014110515536N2. Written informed consent was obtained from all study participants.

### Participants

Women diagnosed with PCOS aged 19–37 years old were identified at their first visit to Arash hospital, Tehran, Iran. Inclusion criteria were as follows: confirmed PCOS based on the 2003 Rotterdam criteria (two of the following features: Oligo-ovulation and/or anovulation, clinical and biochemical hyperandrogenism, and polycystic ovaries in ultrasonography). Women were unsuitable for inclusion if their body mass index (BMI) was below the specified range (BMI < 25), they had a history of thyroid disorders, hyperprolactinemia, Cushing’s syndrome, liver and kidney disease, cardiovascular disease, digestive diseases (food allergies, celiac disease, irritable bowel disease), high blood pressure and diabetes, autoimmune disease, allergy to probiotic capsules or placebo, current or previous consumption of antibiotic, multivitamin mineral supplements, probiotics, perebiotic, synbiotics and specific diet or physical activity programs (within the last 3 months), or they were pregnant or breast-feeding.

### Intervention

Synbiotics and placebo capsules were produced and supplied by Zist Takhmir Company. Each active synbiotics capsule contained 500 mg of seven strains beneficial bacteria (Lactobacillus acidophilus 3× 10^10^ CFU/g, Lactobacillus casei 3× 10^9^ CFU/g, Lactobacillus bulgaricus 5× 10^8^ CFU/g, *Lactobacillus rhamnosus* 7× 10^9^ CFU/g, Bifidobacterium longum 1× 10^9^ CFU/g, Bifidobacterium breve 2× 10^10^ CFU/g, Streptococcus thermophilus) 3× 10^8^ CFU/g) and prebiotic Inulin (fructooligosaccharide). Placebo capsules contained 500 mg starch and maltodextrins without bacteria. Both capsules were identical in appearance, smell and taste, and packaged in compartment labeled as either drug A or drug B, and these capsules were stored at − 5 °C until dispensed to participants. Capsule identification for patients and research staff was not possible. The viability of the synbiotics was confirmed by regular analysis of capsules in the hospital laboratory.

### Randomization and concealment

Allocation to treatment groups was conducted by an independent researcher using a random number sequence, generated with a computer-generated randomization scheme, according to a randomised block design. The block size was six. The randomization list was concealed from the primary researcher who enrolled and assessed participants in sequentially numbered, sealed, opaque envelopes. The study period consisted of a 12-week consumption period, in which initiation of the test and consumption of either the synbiotics or placebo was designated as week 0 (W0). Compliance with the drug consumption guidelines was monitored via phone call once per week. Patients were requested not to alter their diets or normal physical activity and avoid consuming other synbiotics and fermented products during the study. The compliance was also guaranteed by the use of three-day dietary records completed during the study and Nutritionist IV program was used to estimate dietary intake of patients.

### Study outcomes and clinical investigations

Anthropometric measurements, dietary intakes and biochemical indices were evaluated in all participants at baseline and after 12 weeks of intervention in each separate arm. Body weight was measured with a digital scale (model 220; Seca, Hamburg Germany; weighing accuracy of 0.1 kg) with subjects in underwear, in an overnight fasting status, without shoes and in a minimal clothing and after voiding their bladder. Body height was measured in centimeters, standing upright, using a wall mounted stadiometer (model 220; Seca) and the reading was taken to the last completed 1 mm (0.1 cm). BMI was calculated as weight (kilograms) divided by height (meters) squared. Waist circumference (WC) was measured with a flexible tape measure, at the uppermost lateral border of the hip crest (ilium), and was recorded to the nearest millimeter. Hip circumference (HC) measured at the largest posterior extension of the buttocks. Waist hip ratio (WHR) was calculated as waist/height. Trained research staff took all measurements.

A volume of 15 CC blood sample were obtained in Vacutainers after 12–14 h of fasting and sera were separated, processed and stored at − 80 °C for the determination of serum TC, TG and HDL. Fasting plasma TC, TG and HDL were measured by standard techniques. TC was determined by an enzymatic colorimetric method using cholesterol esterase, cholesterol oxidase, peroxidase and the chromagen 4-aminophenazone/phenol. TG levels were determined by an enzymatic colorimetric method using lipoprotein lipase glycerokinase, glycerophosphate oxidase and the chromagen 4-aminophenazone/N-ethyl-N-(3-sulphopropyl)-m-amisidine. HDL was determined by immunoinhibition assay. LDL was calculated according to the Friedewald formula as follow: LDL = TC – HDL − (TG/5). For FSH, LH, E2, Progesterone and Testosterone measurements, immunometric assays based on enhanced luminescence were used (electrochemical luminescence analyzer, E411; Roche Diagnostics, Mannheim, Germany). The results are expressed as IU/L. All samples were analyzed at the Clinical Chemistry Laboratory of the Arash Hospital, Tehran, Iran.

### Sample size and statistical analysis

It was assumed that a total sample of 100 subjects (50) women per group, (which included a 15% dropout factor) would provide 80% power to detect a difference in mean of TG between the synbiotics and placebo groups. Assuming means of 250(mg/dl) in synbiotics group and 190(mg/dl) in the placebo group; a standard deviation (SD) of 100; and a two-sided test having a type I error of 0.05.

All statistical analyses were performed with IBM SPSS software for Windows (version 20.0; IBM). Baseline characteristics were compared among the two intervention groups by using the independent sample t– test for continuous data and a chi-square test for categorical data. The changes in anthropometric measurements, nutrient intakes, and blood lipid parameters of the patients between the beginning and end of the intervention were compared by paired sample t-tests. The magnitude of the effect is presented as mean difference and its 95% confidence interval. Linear mixed effects model were fit to assess changes from baseline within the treatment groups and differences of those changes between treatment groups with respect to continuous outcomes over time. Data were analysed according to the intention-to-treat principle.

## Results

**Fig. 1:**
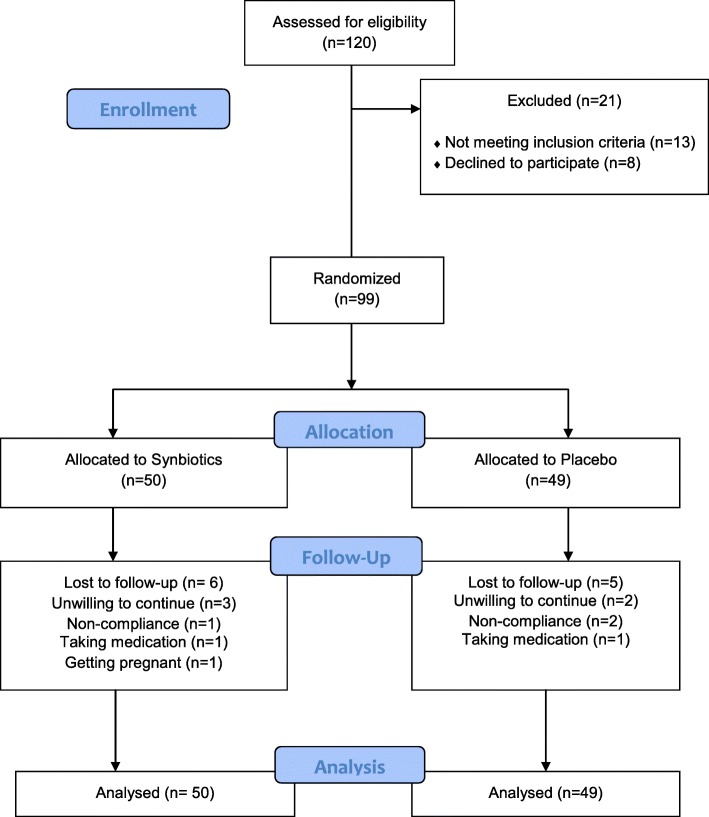
Flowchart showing participants’ recruitment. Non-compliance of the allocated intervention (*n* = 3), taking medication (*n* = 2), unwilling to continue (*n* = 5) and getting pregnant (*n* = 1).The analysis was intention-to-treat approach (ITT)

Recruitment of study participants commenced in September 2015 and ended in July 2016. During two months, 120 women were screened for eligibility; 99 women had inclusion criteria and were randomly assigned to either the placebo or the intervention groups. Eleven patients dropped out during the study period because of non-compliance to the allocated intervention (*n* = 3), no drug intake (*n* = 2), unwillingness to continue (*n* = 5) and pregnancy (*n* = 1). Finally, 88 subjects (synbiotics [*n* = 44], placebo [*n* = 44]) completed the study (Fig. 1). There were no significant differences between the patients of the two groups in maternal age, BMI, marital status, history of previous pregnancy, infertility, history of type2 diabetes, or menstruation (Table 1).

**Table 1:** Patient’s characteristic after random assignment

	Total	Groups		*P*
		Intervention	Control	
Maternal age (years)				
Mean ± SD	28.5 ± 5.3	28.1 ± 5.5	29 ± 5.1	0.471†
Median (range)	28 (19 to 37)	27 (19 to 37)	28 (19 to 37)	
BMI(Kg/m2)				
Mean ± SD	32.46 ± 5.27	32.89 ± 6.11	32 ± 4.23	0.435†
Median (range)	31.54 (28.01 to 35.59)	30.6 (28.01 to 36.16)	31.76 (28.1 to 35.15)	
Marriage				
Single	18 (20.5%)	10 (22.2%)	8 (18.6%)	0.674*
Married	70 (79.5%)	35 (77.8%)	35 (81.4%)	
Widow	0 (0.0%)	0 (0.0%)	0 (0.0%)	
Divorced	0 (0.0%)	0 (0.0%)	0 (0.0%)	
History of previous pregnancy				
Yes	41 (46.6%)	21 (46.7%)	20 (46.5%)	0.988*
No	47 (53.4%)	24 (53.3%)	23 (53.5%)	
Infertility				
Yes	35 (39.8%)	17 (37.8%)	18 (41.9%)	0.696*
No	53 (60.2%)	28 (62.2%)	25 (58.1%)	
History of type2 diabetes				
Yes	17 (19.3%)	9 (20.0%)	8 (18.6%)	0.868*
No	71 (80.7%)	36 (80.0%)	35 (81.4%)	
Menstruation				
Normal	30 (34.1%)	14 (31.1%)	16 (37.2%)	0.834*
Oligoamenorhea	43 (48.9%)	23 (51.1%)	20 (46.5%)	
Amenorrhea	15 (17.0%)	8 (17.8%)	7 (16.3%)	

† Based on t-test. * Based on Chi-Square test

Tables 2 and 3 present baseline, post intervention and values of changes for anthropometric measurements, nutrient intakes, and blood lipid parameters in synbiotics and placebo group, respectively. No significant difference was detected in nutritional intake (average calorie, macronutrients, and fiber intake) from pre intervention to post intervention between synbiotics and placebo groups. No statistically significant differences existed in anthropometric measurements and blood lipid parameters between the synbiotics and placebo groups at baseline.

**Table 2:** Dietary intakes of study participants throughout the study

	Intervention	Control	Diff ^a^	95% CI		*P*
	Mean ± SD	Mean ± SD		Lower	Upper	
Energy intake (kcal)						
Pre	2317.27 ± 542.81	2210.05 ± 360.47	107.22	−88.99	303.43	0.28
Post	2322.6 ± 550.2	2204.8 ± 336.4	117.72	−76.61	312.04	0.344
Change	5.33 ± 66.89	−5.25 ± 54.59	−10.50	− 36.44	15.44	0.423
Carbohydrate intake (% of TE) ^b^						
Pre	52.18 ± 5.09	52.95 ± 4.61	− 0.78	−2.84	1.29	0.457
Post	52.22 ± 4.17	53.28 ± 4.42	−1.06	−2.88	0.76	0.276
Change	−0.04 ± 2.14	− 0.33 ± 2.09	0.28	− 0.62	1.18	0.535
Protein intake (% of TE)						
Pre	11.62 ± 2.38	12.28 ± 2.21	− 0.66	−1.63	0.32	0.183
Post	11.67 ± 2.36	12.3 ± 2.41	− 0.64	−1.65	0.38	0.859
Change	−0.04 ± 1.19	− 0.02 ± 1.26	−0.02	− 0.54	0.50	0.936
Fat intake (% of TE)						
Pre	36.2 ± 4.47	34.81 ± 4.18	1.39	− 0.45	3.22	0.137
Post	36 ± 4	34 ± 4	1.74	0.18	3.30	0.067
Change	0.04 ± 2.22	0.4 ± 1.85	−0.35	−1.22	0.52	0.424
Fiber Intake(g)						
Pre	11.86 ± 1.55	12.19 ± 1.7	−0.33	−1.02	0.36	0.343
Post	12.02 ± 1.52	12.35 ± 1.32	−0.33	−1.02	0.36	0.622
Change	−0.17 ± 0.87	−0.16 ± 0.9	−0.33	−1.02	0.36	0.984

a Intervention minus control group

^b^*TE* total energy

**Table 3:** Comparison of anthropometric measurements and blood lipid parameters between two Groups

	Intervention	Control	Diff ^a^	95% CI		*P*
	Mean ± SD	Mean ± SD		Lower	Upper	
Weight1						
Pre	84 ± 18	80.3 ± 12.1	3.67	−2.87	10.21	0.267†
Post	83.81 ± 18.66	80.23 ± 11.39	3.58	−3.01	10.17	0.891§
Change	0.18 ± 2.34	0.08 ± 8.7	0.09	−2.58	2.77	0.944
BMI1						
Pre	32.89 ± 6.11	32 ± 4.23	0.89	−1.37	3.14	0.435†
Post	32.81 ± 6.33	32.04 ± 4.58	1.19	− 1.60	3.13	0.524§
Change	−0.07 ± 0.91	0.5 ± 3.77	− 0.12	− 1.27	1.02	0.829
Waist circumference						
Pre	90.5 ± 12.3	90 ± 9	0.55	−4.04	5.14	0.814†
Post	89.87 ± 13.03	89.56 ± 8.87	0.32	−4.43	5.06	0.832§
Change	0.65 ± 3.21	0.42 ± 5.43	0.23	−1.65	2.11	0.810
Hip circumference						
Pre	112 ± 10	110.55 ± 8.76	1.72	−2.34	5.79	0.400†
Post	111.59 ± 10.34	110.47 ± 7.48	1.12	− 2.72	4.96	0.66§
Change	0.69 ± 2.67	0.09 ± 5.06	0.60	−1.10	2.31	0.482
WHR1						
Pre	0.81 ± 0.06	0.82 ± 0.07	−0.01	−0.04	0.02	0.478†
Post	0.8 ± 0.07	0.81 ± 0.06	−0.01	− 0.03	0.02	0.705§
Change	0.001 ± 0.03	0.004 ± 0.03	−0.003	−0.01	0.01	0.599
Blood pressure systolic						
Pre	113 ± 25	114 ± 10	−0.88	−9.17	7.41	0.833†
Post	117 ± 12	115 ± 10	1.98	−2.79	6.74	0.28§
Change	−3.46 ± 22.49	−0.6 ± 5.55	−2.86	−9.96	4.24	0.425
Blood pressure diastolic						
Pre	75.67 ± 9.81	73.36 ± 7.99	2.31	−1.52	6.14	0.234†
Post	73 ± 10	74 ± 8	− 1.43	−5.29	2.42	0.025§
Change	2.91 ± 8.14	−0.83 ± 4.79	3.74	0.87	6.62	0.011
Total cholesterol (mg/dl)						
Pre	175.2 ± 27.5	175.1 ± 28.7	0.08	−11.82	11.99	0.989†
Post	170 ± 24	173 ± 32	−3.03	−14.85	8.78	0.433§
Change	5 ± 18.53	1.88 ± 20.76	3.12	−5.22	11.45	0.459
High density lipoprotein cholesterol(mg/dl)						
Pre	46.44 ± 7.69	45.19 ± 8.14	1.26	−2.10	4.61	0.458†
Post	45 ± 8	45 ± 8	−0.55	−3.79	2.70	0.023§
Change	1.71 ± 3.75	−0.09 ± 3.09	1.80	0.34	3.26	0.016
Low density lipoprotein cholesterol(mg/dl)						
Pre	97 ± 19	96 ± 20	0.80	−7.52	9.12	0.849†
Post	92 ± 19	95 ± 20	−3.87	−11.99	4.26	0.038§
Change	−5.27 ± 11.32	0.6 ± 9.63	4.66	0.20	9.13	0.041
Triglyceride(mg/dl)						
Pre	139 ± 78	134 ± 87	4.92	−29.97	39.81	0.78†
Post	141 ± 78	130 ± 83	10.53	−23.56	44.63	0.469§
Change	−2.24 ± 44.59	3.37 ± 41.48	−5.62	−23.89	12.66	0.543

^a^ Intervention minus control group

† Based on t-test

§ Based on Linear mixed effects model (the included variables were: basic value of dependent variable, treatment type, BMI and Maternal age)

Results of linear mixed effects models showed statistically significant differences between the two groups in LDL (*P* = 0.041) and HDL (*P* = 0.016) at the end of study, adjusted for BMI, maternal age, and baseline values. Consumption of a synbiotics, compared to the placebo, resulted in a significant decrease in LDL value (Change Mean Difference: 4.66, 95%CI: 0.20, 9.13) and a significant increase in HDL (Change Mean Difference: 1.80, 95%CI: 0.34, 3.26). Although we failed to find a significant effect of synbiotics consumption on TC (Change Mean Difference: 3.12, 95% CI: − 5.22, 11.45, *P* = 0.459) and TG (Change Mean Difference: -5.62, 95% CI: − 23.89, 12.66, *P* = 0.543) levels. We did not find differences in weight (Change Mean Difference: 0.09, 95% CI: − 2.58, 2.77, *P* = 0.944), BMI (Change Mean Difference: -0.12, 95% CI: − 1.27, 1.02, *P* = 0.829), WC(Change Mean Difference: 0.23, 95% CI: − 1.65, 2.11, *P* = 0.810), HC (Change Mean Difference: 0.60, 95% CI: − 1.10, 2.31, *P* = 0.482) or WHR (Change Mean Difference: -0.003, 95%CI: − 0.01, 0.01, *P* = 0.599) between groups.

## Discussion

The results of current study showed that synbiotics supplementation for 12 weeks can increase HDL and decrease LDL in patients with PCOS. This effect was also significant after adjusting for confounding variables, including gestational age, BMI and baseline indices. However, no significant between-group difference was found for anthropometric indices such as BMI, WC, HC and WHR in women with PCOS.

In line with the results of this trial, a recent 12-week clinical trial among 60 women aged 18–40 years old diagnosed with PCOS demonstrated that the administration of one serving/day of synbiotics capsule (500 mg), resulted in significant decreases in TG, VLDL, while no alterations in anthropometric indices were reported. In the mentioned study, three probiotic strain was similar to the present study (Lactobacillus acidophilus strain T16 (IBRC-M10785), Lactobacillus casei strain T2 (IBRC-M10783), and Bifidobacterium bifidum strain T1 (IBRC-M10771) (2 × 109 CFU/g each)) [21]. On the contrary, Ahmadi et al. showed reduction effects of probiotic on weight and BMI in patients with PCOS. In this randomized, double-blind, placebo-controlled trial, 60 women with PCOS were randomized to receive probiotic capsule (*n* = 30) or placebo (*n* = 30) for 12 weeks. In their study, dry and freeze probiotics of Lactobacillus acidophilus, Lactobacillus casei and Bifidobacterium bifidum were used. Compared to the present study, a more limited range of probiotics was used and prebiotic was not used [14]. Furthermore, a 2019 meta-analysis (*N* = 8 trials, nine treatment arms), reported beneficial effects of pro−/synbiotics supplementation on insulin resistance indices without any significant effects on anthropometric measurements [22]. In a systematic review and meta-analysis, Hadi et al. evaluated the effects of synbiotics supplementation on anthropometric indices among participants with overweight or obesity. This meta-analysis of 23 randomized trials indicated that supplementation with synbiotics can decrease body weight and WC. In contrast, synbiotics did not have favorite effects on BMI and body fat compared with the placebo group [23]. A study by Gomes et al. in 2017 showed that probiotic supplement reduces abdominal fat in women with obesity and overweight. In this study, a probiotic mixture, without prebiotics, was used. The probiotic composition of the Gomez’s study included Lactobacillus acidophilus, Casei Lactobacillus, Lactococcus lactis, Bifidum bifidobacterium, Bifidobacterium lactis, which is different from the probiotic composition of the present study. Also, the study was conducted on women with obesity who received diet alongside probiotics [24]. Another study revealed that the synbiotics supplementation reduces weight and improves the BMI in people with diabetes. In this study, the probiotic mixture of the Lactobacillus family, the Bifidobacterium family and Streptococcus thermophiles was used. Prebiotics consisted of freto-oligosaccharides and lactose, as well as vitamin group B supplement, maltodextrin and magnesium. Different compound of probiotic and prebiotic supplements could lead to the difference between findings of this study and the present study [25]. In a systematic review study of clinical trials, it was shown that probiotic supplement could cause weight loss and improve BMI when a variety of strains was used. The supplement also more affected people with overweight. The duration of supplement should be more than 8 weeks [26]. Ferolla et al. in 2016 showed that probiotic supplement could lead to weight loss in patients with nonalcoholic steatohepatitis (NASH). They also used *Lactobacillus reuteri* strain as probiotic supplement, and guar gum and inulin as prebiotic supplement. Diet was also prescribed for all participants [27]. In a systematic review study, Maria Jose Sáez-Lara et al. evaluated the effects of synbiotics supplementation on the prevention and treatment of obesity, IRS, DM2, and non-alcoholic fatty liver disease (NAFLD). Supplementations of synbiotics and probiotic were associated with weight loss and BMI in people with obesity. This effect has been observed in different strains and in different prebiotic combinations [28]. Bernini, L.J.a et al. in 2016 showed that supplement of Lactobacillus lactis strain decreases BMI in patients with metabolic syndrome. In their study, the probiotic strain as fermented milk was consumed by participants. The length of the study was 45 days [29]. It seems that some divergence between these trials described in these two reviews is potentially due to differences in the probiotic strain, study duration and severity of disease.

The present study showed that synbiotics supplement could reduce LDL and increase HDL in patients with PCOS. Although these changes were statistically significant, but their values are not clinically significant, and it seems that synbiotics may have complementary therapies role in LDL and HDL managements. Previous studies have confirmed a decrease in LDL and increased HDL through probiotic supplement [30]. Decreased LDL and TC and TG have been observed in previous studies [31]. In a clinical trial on men and women with hypercholesterolemia, a significant decrease was observed in LDL and TC after a 12-week probiotic and prebiotic supplement [32]. A systematic review investigated the use of probiotics and prebiotics simultaneously and their effect on lipid profile. Synbiotics supplement effectiveness is better than probiotic effectiveness only in improving lipid profiles [33, 34]. In addition to the combination of probiotics and prebiotics, the probiotic strain also may be effective in reducing serum cholesterol. Another systematic review and meta-analysis conducted in 2015 showed that the strain of Lactobacillus Acidophilus had the highest effect on lowering LDL compared to other probiotic strains [35]. Bernini et al. found that probiotic supplement reduced LDL and TC [29]. In a systematic review study in 2011, it was concluded that probiotic supplement decreased TC and LDL. The suggested mechanism in this study was to integrate cholesterol into the cell wall of probiotic bacteria in the intestinal tissue and to prevent the absorption of cholesterol [36, 37]. Another suggested mechanism of probiotic supplement for the reduction of cholesterol was production of hydrolytic enzymes by these microorganisms. It causes deconjugation and reduction in absorption of bile acids in the intestine; thereby eliminating the acids through feces and consequently, reducing cholesterol levels [38]. On the other hand, probiotics in the intestine increase the Short-chain fatty acids (SCFA), such as acetate and propionate. Propionate has a variety of metabolic effects and in the liver, inhibits the enzyme HMG-COA reductase, a restriction enzyme in the production of cholesterol in the body. In this way, probiotics supplement may also reduce total cholesterol and LDL levels [39].

The strengths of the study are the inclusion of all PCO phenotypes, which allowed a much greater possibility of drawing generalizable conclusions and small number of participants’ drop out. However, this trial has several limitations that need to be addressed. First, lack of an objective method for measuring patient compliance like fecal bacterial profiles. Second, probability of recall bias due to dietary intake was assessed by using 3-day food records. Third, it would be better to compare four groups: placebo, synbiotic alone, probiotic alone, and the combination of probiotic and synbiotic, but we have resource limitation to conduct this design. And finally maybe longer duration could make us able to find more reliable and clinical worthwhile outcomes.

## Conclusions

The present study showed that the use of synbiotics in reducing LDL and also increasing HDL in women with PCOS could be effective. Considering that lipid profile disorders are one of the common problems in PCOS patients, it seems that synbiotics supplement in these patients could reduce the symptoms of the disease. Based on this initial study, 12 weeks of synbiotics supplementation among PCOS women resulted in beneficial effects on LDL and HDL. The clinical impact - both short term and long term - is unknown. It is recommended that more clinical studies with a larger sample size be conducted in the future.

## Data Availability

The data that support the findings of this study are available from the corresponding author upon reasonable request.

## Abbreviations

PCOS: Polycystic ovary syndrome
LDL: Low-density lipoprotein cholesterol
CMD: Change Mean Difference
HDL: High-density lipoprotein cholesterol
TC: Total cholesterol
TG: Triglyceride
WC: Waist circumference
HC: Hip circumference
WHR: Waist hip ratio
SD: Standard deviation
BMI: Body mass index
NASH: Nonalcoholic steatohepatitis
NAFLD: Non-alcoholic fatty liver disease
